# Use of contrast-enhanced MRI of the heart in detection of risk of supraventricular tachyarrhythmias in patients with recent myocardial infarction

**DOI:** 10.1186/1532-429X-17-S1-P255

**Published:** 2015-02-03

**Authors:** Olga Mochula, Tatiana Shelkovnikova, Wladimir Y Ussov, Vadim E Babokin, Sergey Popov

**Affiliations:** Federal State Budgetary Scientific Institution “Research Institute for Cardiology”, Tomsk, Russian Federation

## Background

Currently, the use of multispiral cardiac X-ray and MRI tomography techniques has been accepted as the most promising approaches both in location and prediction of substrate of supraventricular arrhythmias both in coronary and non-coronary patients.

## Methods

Twenty persons (all males but two, as old as 57 ± 7 years) with recent (< 4 months) myocardial infarction of left ventricle were included. Paramagnetic contrast-enhanced T1-weighted SE (TR=450-600 ms, TE=15-20 ms) MRI of the heart has been employed for visual detection of risk as well of anatomic locations of atrial arrhythmias in twenty patients with previous myocardial infarction. Contrast enhancement was carried out using intravenous injection of widely available extracellular paramagnetics in a dose as high as 2 ml of 0,5 M solution per 10 kg of body mass (BM). Two groups were separated in accordance with the electrophysiologic and clinical data. The Group 1 included eight persons without supraventricular tachycardias (SVT) in the course of follow-up for four months, and the Group 2 comprised twelve patients with frequent SVT paroxysms, all were later suggested for maze procedure. Also eight persons were recruited to the control group, all without cardiac pathology.

## Results

In the persons of the control group the index of enhancement (IE) of the T1-w. SE MRI of the atrium was essentially uniform over the atrial wall and did not exceed 1,03±0,4 anywhere.

The MRI data delivered highly significant differences between the groups in average values of atrial volume (in Group 1 56,8±9,1 cm^3^, whereas 91,3±10,0 in the Group 2, p<0,02). The IE, calculated for the site of most prominent uptake of contrast, also differed significantly (1,2±0,02 in Group 1 and 1,4±0,07 in Group 2, p < 0,005), as well as the overall volume of contrast uptake (0,3±0,09 cm^3^ in the Group 1 and 0,8±0,2 in the Group 2, p<0,05). When anatomically placing electrophysiologic data over the map of uptake of paramagnetic over the atrium, the electrophysiologic substrate of the arrhythmia was located in ten cases of twelve closely to the region of most intensive uptake of paramagnetic to the atrial wall.

No significant correlation has been revealed between the particular indices both in the groups and in the overall population of the study.

It has been shown that pathologic uptake of paramagnetic to the myocardium of atria with index of enhancement over 1,27 (in T1-weighted spin-echo mode) with concomitant increase of left atrial volume over 75 ml provide a prognostic factor for the nearest future manifestation of atrial tachyarrhythmias and endocardial destruction procedure.

## Conclusions

Therefore the contrast-enhanced MRI of the heart can be routinely employed as effective tool of selection of persons with high risk of supraventricular tachyarrhythmias among patients with ischaemic heart disease and previous myocardial infarction.

## Funding

N/A.

**Figure 1:**
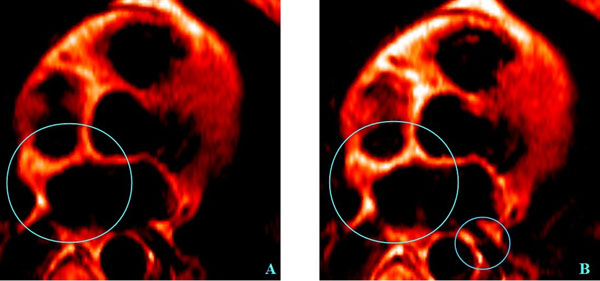
T1-weighted spin-echo CE MRI of the heart I a patient with a recent myocardial infarction and currently frequent SVT paroxysms. A - T1-w. SE MRI of the heart, with the axial slice position over the atrium, acquired before injection of paramagnetic contrast. B - T1-w. SE MRI of the heart in the same case, with the same position of the axial slice over the atrium, acquired injection of 15 ml of 0,5M paramagnetic contrast (as 2 ml per 10 kg of BM). The big circle depicts pathologic uptake of contrast to the atrial septum, ostium of the right pulmonary vein and interventricular septum, the little circle depicts the pathologic uptake of contrast to the ostium of the left pulmonary vein.

